# Brain Activation of Identity Switching in Multiple Identity Tracking Task

**DOI:** 10.1371/journal.pone.0145489

**Published:** 2015-12-23

**Authors:** Chuang Lyu, Siyuan Hu, Liuqing Wei, Xuemin Zhang, Thomas Talhelm

**Affiliations:** 1 Beijing Key Lab of Applied Experimental Psychology, School of Psychology, Beijing Normal University, Beijing, China; 2 State Key Laboratory of Cognitive Neuroscience and Learning & IDG/McGovern Institute for Brain Research, Beijing Normal University, Beijing, China; 3 Center for Collaboration and Innovation in Brain and Learning Sciences, Beijing Normal University, Beijing, China; 4 Booth School of Business, University of Chicago, Chicago, Illinois, United States of America; University Medical Center Goettingen, GERMANY

## Abstract

When different objects switch identities in the multiple identity tracking (MIT) task, viewers need to rebind objects’ identity and location, which requires attention. This rebinding helps people identify the regions targets are in (where they need to focus their attention) and inhibit unimportant regions (where distractors are). This study investigated the processing of attentional tracking after identity switching in an adapted MIT task. This experiment used three identity-switching conditions: a target-switching condition (where the target objects switched identities), a distractor-switching condition (where the distractor objects switched identities), and a no-switching condition. Compared to the distractor-switching condition, the target-switching condition elicited greater activation in the frontal eye fields (FEF), intraparietal sulcus (IPS), and visual cortex. Compared to the no-switching condition, the target-switching condition elicited greater activation in the FEF, inferior frontal gyrus (pars orbitalis) (IFG-Orb), IPS, visual cortex, middle temporal lobule, and anterior cingulate cortex. Finally, the distractor-switching condition showed greater activation in the IFG-Orb compared to the no-switching condition. These results suggest that, in the target-switching condition, the FEF and IPS (the dorsal attention network) might be involved in goal-driven attention to targets during attentional tracking. In addition, in the distractor-switching condition, the activation of the IFG-Orb may indicate salient change that pulls attention away automatically.

## Introduction

Multiple object tracking (MOT) is an effective method used in visual cognitive processing studies of dynamic scenes. Researchers have conducted many studies to investigate the cognitive mechanism behind MOT and multiple identity tracking (MIT) since Pylyshyn and Storm [1] first established the MOT paradigm. MOT tasks focus on visual attention in early cognitive processing, whereas MIT focuses on later cognitive processing (e.g., perception and visual short-term or working memory) [1–10]. In recent studies, researchers have paid more attention to how observers dynamically track, perceive, and memorize multiple identities.

MIT studies have separated the identity encoding system and the positional encoding system [3]. Behavioral results indicate that people’s identity tracking performance with familiar targets is much better than with unfamiliar targets [5,10,11]. By comparing the tracking of familiar objects to unfamiliar objects, researchers have found that MIT is a two-stage process, with one stage for location processing and the other stage for identity processing [11]. The study showed that when tracking unfamiliar targets, regions that negotiate with the goal-directed attention network are activated (middle frontal gyrus, the precentral gyrus, and the right insular cortex), and when tracking familiar objects, regions connected with the function of increased memory, naming, and parts of the “resting state” network are activated [11]. These results indicate that unfamiliar targets require more attentional resources for processing the identities of objects. Studies have also found the dissociated processing of identity and location in a static working memory task (n-back task). It shows that verbal distraction can impair object visual working memory and that motion distraction can interfere with spatial working memory [12], which indicates dissociated processing of identity and location.

However, the tracking of location and identity are not completely isolated in MIT [13]. Observers are able to bind the correct identities to corresponding spatial locations dynamically. There appears to be a trade-off between location tracking and identity binding [10,14]. A less resource-demanding identity processing would lower binding load and then enhance tracking performance [15], such as with familiar objects [10]. However, Oksama and Hyönä suggested that there is a temporary episodic buffer for identity-location binding [5]. The average capacity of the buffer for binding might be four [5], which corresponds to the average capacity of location tracking. The bindings may be further used for tracking and retrieving identities and locations [5]. Therefore, the efficiency and quality of identity and location encoding will influence identity-location binding [5]. This indicates that, when tracking complex and unfamiliar objects, identity-location binding is less effective or cohesive than tracking simple and familiar objects.

If targets switch identities during tracking, it could force viewers to refresh, or rebind the features and locations [16]. In static visual tasks, feature binding can be easily achieved, such as in a probe-change detection task [17]. Static visual research on letter-location binding has found that the fronto-parietal network plays an important role in feature binding [18,19]. However, probe-change detection affects the results of binding in terms of whether participants can detect the change of probed objects. Thus, we used an identity-switching version of the MIT task to observe brain activation during attentional tracking after identity switching during tracking. Three identity-switching conditions were conducted in this study: a target-switching condition, a distractor-switching condition, and a no-switching condition. In the target-switching condition, the identities of targets switched with each other while moving. In the distractor-switching condition, the identities of distractors switched. A typical MIT task without identity switching was used as the baseline.

In order to keep tracking targets, people need to pay more attention to targets in the target-switching condition because the targets switch identities. Researchers have studied the brain regions responsible for attentional tracking of targets and the neural mechanisms of MOT. By adding additional tracking items to increase attentional load [20,21], researchers have found two separate areas of activation: one area increases with attentional load, and the other remains stable. The first area is mainly located in the parietal lobe [20–22] and is essential in visual attentional tracking [23]. More specifically, the posterior intraparietal sulcus (PIPS) has been suggested to function as a spatial index or spatial tag [20–25] that points at the locations of attended targets. In addition, the anterior intraparietal sulcus (APIS) represents the information about the objects [25,26]. The stable part of activation includes the superior parietal lobe (SPL) and FEF [26], which are related to task functions that do not vary with attentional load, such as suppression of eye movement [20,24,26].

While participants need to increase their attention to targets in MOT tasks, they need to inhibit their attention to distractors [6–8,27]. However, the neural mechanism behind inhibition to distractors is still unclear. In the task used in this study, novel items and salient changes attract attention [28]. The salience of the stimulus plays a large role in determining visual selection in the first 150 ms, but later (> 150 ms) visual selection shifts to task-related targets [29]. And top-down attention control helps people disengage their attention from distractors [29].

In contrast to this view, Anderson and Folk suggest that location-specific inhibition does not necessarily capture attention that early (< 150 ms) [30]. Instead, they argue that the process of visual selection and inhibition might simultaneously influence people’s perception, and whichever is stronger will determine the outcome [30]. Thus, the target-switching condition might tend to enhance attention to targets. Meanwhile, in the distractor-switching condition, brain activation could show (1) location-specific inhibition or (2) attention shift from distractors.

The difference between the target-switching condition and the no-switching condition is that the identity of targets switches in the target-switching condition. We predict that regions responsible for attention focused on targets will be activated. The difference between the distractor-switching condition and the no-switching condition is that, in the distractor-switching condition, the identity of the distractors switches. Thus, compared to the no-switching condition, we predict that the regions responsible for attentional inhibition or attentional shift to targets will be activated. Furthermore, the comparison between the target-switching condition and the distractor-switching condition will help reveal how people distribute their attention.

## Materials and Methods

### Participants

Nineteen right-handed (mean age = 22, age range: 18–25 years, 8 females) undergraduates were recruited from Beijing Normal University. All participants had normal or corrected-to-normal visual acuity. The data from four additional participants was excluded: one because of missing behavioral results, one because of low behavioral performance, and two because of technical problems during fMRI data acquisition. All participants were provided thorough instructions for the experiment, and they received 20 practice trials before the experiment to become familiar with the task.

### Ethics Statement

All observers provided written informed consent prior to the experiment. The study was approved by the Institutional Review Board of the National Key Laboratory of Cognitive Neuroscience and Learning, School of Brain and Cognitive Sciences at Beijing Normal University.

### Stimuli

The program for the experiment used Microsoft Visual Basic.NET (version 2013) running on a Core i5 laptop. The “stopwatch” function was used to achieve a precision of 1 millisecond. Stimuli were projected onto a translucent screen placed at the back of the magnet bore. Participants viewed the screen through a mirror at a distance of ~30 cm from the eyes. The background color of the task was dark grey (RGB (64, 64, 64)). The motion of stimuli appeared smooth and continuous on this display (1024×768 pixel, a pixel approximately equal to 0.032 cm).

**Fig 1:**
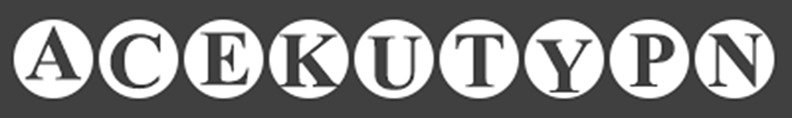
The stimuli used in the experiment. The dark grey background of this picture is to highlight the rim of each white circle.

All stimuli were presented within a 28.72°× 21.74° rectangle motion area with a white border (0.12° width, RGB (255,255,255)). A white fixation (0.73°×0.73°) was placed in the middle of the rectangle. The stimuli were eight solid white circles with a diameter of 2.44° and a letter (the letters are “A”, “C”, “E”, “K”, “N”, “P”, “Y”, “T”, and “U”) in the middle of each circle (Fig 1). These stimuli were selected randomly in trials and randomly marked as targets or distractors. For example, the letter “A” could be one of the targets in one trial, a distractor in another trial, or not selected. In addition, these monosyllabic letters were used to avoid number-stimuli that would activate the parietal lobe [31,32]. College students majoring in psychology rated the letters for shape resemblance. The nine letters with the lowest shape resemblance ratings were chosen for the experiment.

### Procedure

**Fig 2:**
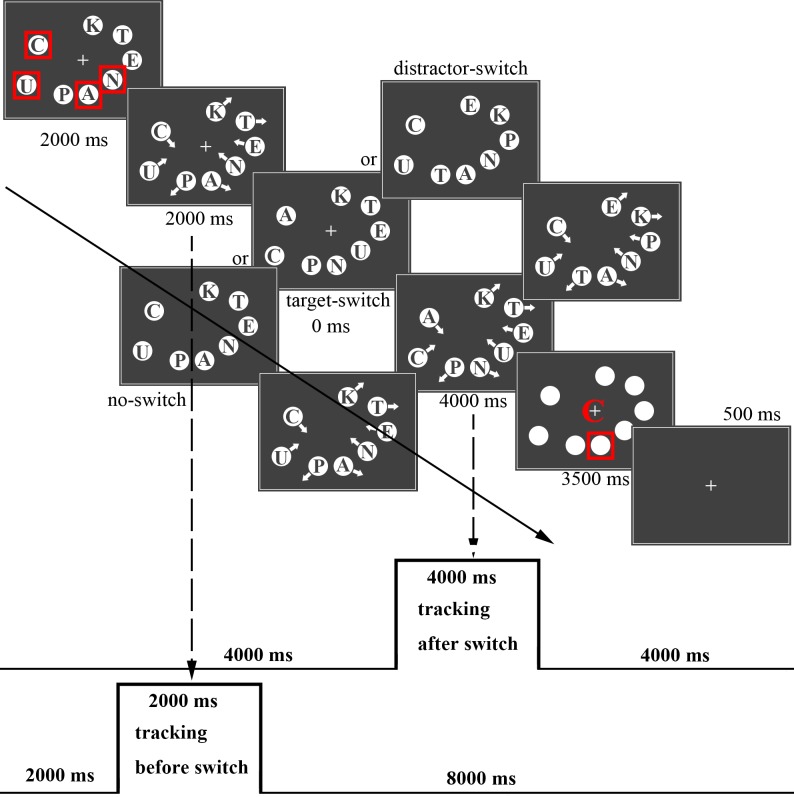
Sample illustrations of a trial.

At first, the objects were distributed randomly in the motion area without overlapping. The letters on them were also randomly selected. Four of the objects were surrounded by a red rectangle (1.47° width, RGB (255, 0, 0)) to indicate targets. The others not surrounded by the red rectangle were labeled as distractors. After 2,000 ms, each object was given an initial speed of 9.37°/s. The objects moved with a 5% probability change of speed, which changes within 5°/s to 13.75°/s. The switch-conditions occurred after tracking for 2,000 ms, and switching was completed immediately. After switching, the objects continued to move for 4,000 ms. Letters were masked by white circles when the object motion stopped. Then one of the targets was surrounded by a red rectangle, and simultaneously a letter (1.47°×1.47°) appeared in the middle of the screen (to prevent potential visual afterimages). The observers were asked to judge whether the specific letter in the middle of the screen was the same letter as the surrounding target. If the probed letter was the identity of surrounding target, they were to press “1” using the left finger; if not, they were to press “2”. If no response was received within 3,500 ms, the trial would be labeled “null”. (See the sample trial procedure in Fig 2.)

The experiment had three conditions (distractor-switching condition, target-switching condition, no-switching condition). In the target-switching condition, the letters of the targets were rearranged among targets, and the new letter on each target was different from the old letter. In the distractor-switching condition, the letters of the distractors were rearranged among distractors, and each distractor had a new letter distinct from the old letter. In the no-switching condition, the letters on the objects did not change during tracking. Thus, the comparison of the target-switching condition and the no-switching condition will show which regions are activated for switching identities. Furthermore, the comparison between the distractor-switching condition, and the no-switching condition will reveal the areas involved in inhibition of distractors.

Each condition consisted of 30 trials. There were 90 trials in total that were organized into nine blocks, each consisting of 10 trials. Each trial lasted for 12 seconds. Observers rested for 24 seconds after each block. The whole experiment lasted approximately 30 minutes. Before scanning, the observers were trained for 20 trials to ensure that they understood the instructions.

A balanced design was used to counterbalance the order effect of the three conditions. The three conditions (distractor-switching condition labeled “D”; target-switching condition labeled “T”; no-switching condition labeled “N”) could be permutated into six types of sequences: “TDNDNTNTD”, “DNTNTDTDN”, “NTDTDNDNT”, “TNDNDTDTN”, “DTNTNDNDT”, and “NDTDTNTND”. The correct answer of the trials was also balanced. Participants had to press the “1” key in 50% of all trials and “2” in the other 50% of trials.

### fMRI data acquisition

fMRI scans were acquired on a 3T scanner (Siemens Magnetom Trio, A Tim System). A standard 12-channel head coil was used. A high-resolution T1 weighted MPRAGE anatomical scan was acquired for registration purposes from each participant (TR = 2300, TE = 2.86, flip angle = 9°, 144 slices, matrix dimensions = 256 × 256, and voxel size = 1 × 1 × 1.33 mm^3^). Functional scans were acquired with a gradient-echo single-shot echo planar imaging sequence (33 slices, interleaved slice order, matrix dimensions = 64 × 64, TR = 2000 ms, TE = 30 ms, flip angle = 90°, FOV = 200 × 200 mm^2^, voxel size = 3.125 × 3.125 × 4 mm^3^), covering the entire brain.

### Statistical analysis of the fMRI data

#### First-level analysis

MRI data were analyzed using SPM8 (Wellcome Department of Imaging Neuroscience, University College London, UK, http://www.fil.ion.ucl.ac.uk/spm) implemented in MATLAB R2013b (MathWorks, Inc., Natick, MA). We performed head motion correction, spatial normalization, and spatial smoothing with a 6 mm full-width at half-maximum Gaussian kernel. The co-registered functional and anatomical images were registered to MNI space (Montreal Neurological Institute) with a resolution of 3 × 3 × 3 mm^3^.

The evoked hemodynamic responses to tracking after switching (4000 ms after switching), and tracking before switching (2000 ms before switching) under three switching conditions, in trials with correct response, were modeled for each subject with a box-car function (Fig 2). Nuisance regressors consisting of the six head motion regressors from the SPM realignment procedure, trials with no response and trials with wrong response were added to the model. At the subject level, three contrast analyses were conducted: target-switching condition vs. distractor-switching condition, target-switching condition vs. no-switching condition, distractor-switching condition vs. no-switching condition. The target-switching condition induced a refresh of identity and location tracking. We assume that, when people are tracking in the target-switching condition, they will focus more attention on targets and inhibit their attention to distracters. In the distractor-switching condition, the switch of identities will draw attention to distractors and then attention shifts to targets. Thus, the comparison of the target-switching condition and the distractor-switching will show which regions are responsible for attention to targets and attention shifts to targets. The comparison between the target-switching condition vs. the no-switching condition will show which regions are responsible for attention to targets and visual analysis. Finally, the comparison of the distractor-switching condition vs. the no-switching condition will show which regions are responsible for attentional inhibition to distractors and attention shifts to targets.

#### Second-level analysis

After all individual data were processed, individual participants’ contrast maps were combined by a one-sample *t*-test for each contrast of interest. The corrections for multiple comparisons (using AlphaSim correction) were confined within the whole-brain mask (size: 53,468 voxels) and were determined by Monte Carlo simulations [33] that were performed using the AlphaSim program in REST[34] (www.restfmri.net). The statistical threshold was set at *p =* 0.05, cluster size > 600 voxels, and edge connected, which corresponds to a corrected threshold of *p =* 0.05. And the XjView software (http://www.alivelearn.net/xjview) was used to provide anatomical labeling of clusters. The results and the anatomical locations were visualized with the MNI Space Utility (MSU; http://www.ihb.spb.ru/~pet_lab/MSU/MSUMain.html) and WFU_PickAtlas (http://www.fmri.wfubmc.edu/cms/software). Significance maps were then projected onto the inflated cortical surface of a standard brain provided by the BrainNet Viewer [35] (http://www.nitrc.org/projects/bnv/) program for display purposes.

#### ROI analysis

To keep tracking targets, participants need to pay attention to targets. We predicted that changes in the target-switching condition might tend to enhance attention to targets comparing to that in the distractor-switching condition. Thus we defined regions for their involvement in the attention networks. The FEF and Intraparietal Sulcus (IPS) are two major parts of dorsal attention network, which is responsible for top-down control of attention in static tasks [36–41]. The inferior frontal gyrus (IFG) is involved in stimulus-driven visual attention as a part of the ventral attention network [36,38,42,43]. In our experiment, “item switching” would certainly raise more attention demand. We defined attention-related regions by comparing “tracking after switch” vs. “tracking before switch” conditions.

We modeled the evoked hemodynamic responses to tracking before switching (all three experimental conditions were included) along with tracking after switching (all three experimental conditions were included; “tracking after switch” and “tracking before switch”, Fig 2). Based on the contrast “tracking after switch” vs. “tracking before switch” (detailed results in S1 Fig and S2 Table), we defined three attention-related regions of interests in each hemisphere: FEF, IPS, and IFG-Orb [20,21,26]. Each ROI was defined as a sphere, which was grown around the peak activation coordinates (10 mm radius) with a threshold of *p* = 0.05 (FDR corrected, two-tailed), and then projected back for each participant and each hemisphere separately. Percent signal change (% SC) data from each ROI was extracted using the MARSBAR toolbox (http://marsbar.sourceforge.net/). The percent signal changes, one per experimental condition per ROI per participant, were then submitted to a 6 (ROIs: left IFG-Orb, right IFG-Orb, left IPS, right IPS, left FEF, right FEF) × 3 (experimental conditions: target-switching, distractor-switching, no-switching) within-subject ANOVA for further analysis. Moreover, pairwise comparisons (Bonferroni corrected) were conducted to find differences of percent signal change in experimental conditions of each ROI.

## Results

### Behavioral results

The mean accuracy rates (mean of the percentage of the correct answer to the whole trials) were above 80% for the three conditions (Table 1 and S1 Table). One participant was excluded because of low behavioral performance (56.7% accuracy in the target-switching condition, 66.7% in the distractor-switching condition, and 56.7% in the no-switching condition). In addition, only 0.994% trials did not receive responses.

**Table 1:** Descriptive statistical results of behavioral data.

		T	D	N
Reaction Time	Medium	1598.5	1662.2	1582.5
	Range	1261.1–2012.1	1334.4–2490.9	1376.5–2052.2
	Mean	1623.9	1649.2	1652.9
	S.D.	210.7	267.6	206.1
	Skewness	0.12	1.57	0.64
	Kurtosis	-0.57	4.68	-0.77
Accuracy rate	Medium	0.833	0.867	0.867
	Range	0.633–0.967	0.567–1.000	0.633–0.967
	Mean	0.821	0.840	0.837
	S.D.	0.104	0.122	0.099
	Skewness	-0.60	-0.87	-0.47
	Kurtosis	-0.67	0.48	-0.86

T = target-switching condition; D = distractor-switching condition; N = no-switching condition. S.D. = standard deviation.

Normality tests were performed on reaction time (RT) and accuracy rates (ACY) with the one-sample Kolmogorov-Smirnov Test. The results show that the distributions in the target-switching condition (RT: *p* = 0.994, ACY: *p* = 0.579), distractor-switching condition (RT: p = 0.674, ACY: *p* = 0.681), and no-switching condition (RT: *p* = 0.716, ACY: *p* = 0.448) fit the normal distribution.

Repeated-measures ANOVA was used to analyze the accuracy rates and RTs. The main effect of the switching condition was not significant for either accuracy rates *F*_(2, 36)_ = 0.408, *p* = 0.668 or RTs *F*_(2, 36)_ = 0.294, *p* = 0.747.

The moderate accuracy rates and RT were consistent with previous research [44]. Each participant received training before scanning to ensure they understood the task and could have stable tracking performance. In addition, since we require participants to answer probe questions as correctly as possible, and participants need to recall the identity of specific targets, it likely took participants more time to ensure that their answers were correct than in other typical MOT tasks.

### Group activation maps

**Fig 3:**
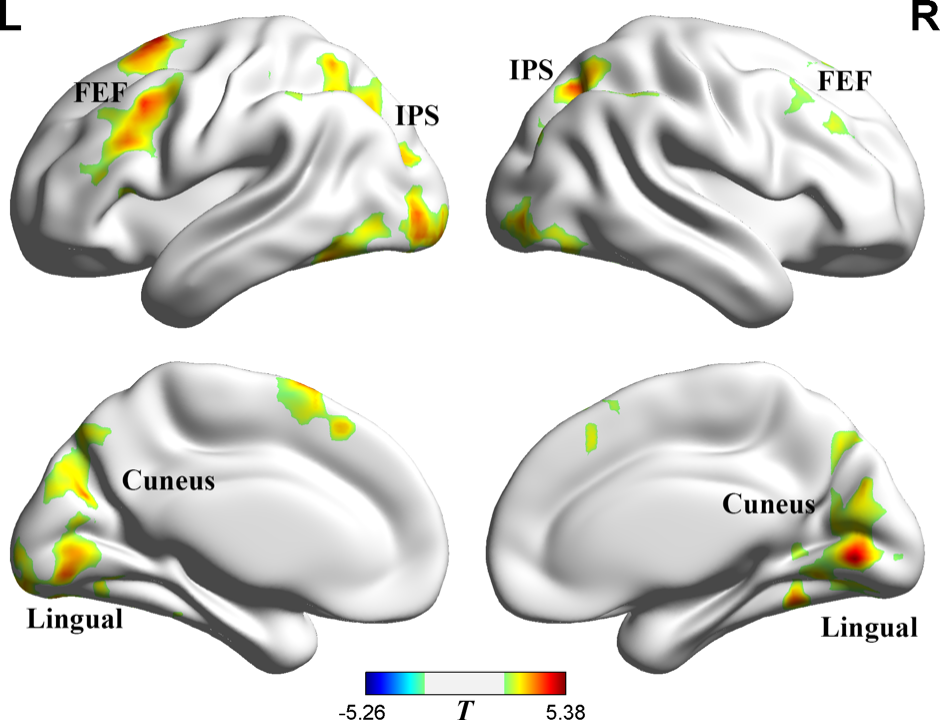
Target-switching condition > distractor-switching condition. Posed on medium view. Threshold: *p* = 0.05 (AlphaSim corrected). The red shows the regions that are more active in the target-switching condition.

Switching identities in the target-switching condition breaks the correspondence relationship of location and identity. However, in the distractor-switching condition, the identity switching of distractors might be inhibited. We compared the target-switching condition with the distractor-switching condition, and found significant (*p =* 0.05, AlphaSim corrected) differences in the frontal eye fields (FEF), the intraparietal sulcus (IPS), visual cortex (Fig 3 and Table 2; target-switching condition > distractor-switching condition). Previous studies have found that the activation of the IPS increases with the attention load of targets during tracking [20,21,26]. The IPS and FEF belong to the dorsal attention network, which has been shown to be closely related to top-down attentional control [36–41]. The FEF has also been reported to modulate the response of the extrastriate cortex [45].

**Table 2:** Region activation in switch conditions contrasts.

Region	Lat.	BA	*x*	*y*	*z*	Vox.	*T*
**I.Target-switching condition > Distractor-switching condition**							
Frontal Eye Fields	L	6,8,9,44	-18	10	67	1021	4.81
	R	6,8,10,48	30	15	27	544	4.84
Intraparietal Sulcus	L	7,40	-31	-56	58	458	3.69
	R	7,40	36	-59	55	359	4.12
Visual cortex	L/R	17,18,19	6	-72	0	3120	5.07
**II.Target-switching condition > No-switching condition**							
Frontal Eye Fields	L	6,8,9,44	-30	10	58	933	6.16
	R	6,8,9	40	10	48	760	3.79
Inferior Frontal Gyrus (pars Orbitalis)	L	47,48	-39	25	-1	702	4.35
	R	47,48	54	22	-3	383	6.08
Intraparietal Sulcus	L	7,40	-22	-69	40	228	5.54
	R	7,40	39	-57	54	289	4.13
Visual cortex	L/R	17,18,19	6	-63	-2	3026	5.64
Middle Temporal Lobule	L	21,48	-47	-40	9	519	3.71
Anterior Cingulate Cortex	L/R	24	-8	4	30	142	4.44
**III.Distractor-switching condition > No-switching condition**							
Inferior Frontal Gyrus (pars Orbitalis)	L	47,48	-33	24	-3	394	5.53
	R	47,48	36	33	-9	319	5.65

Threshold: *p* = 0.05 (AlphaSim corrected). x, y, and z refer to coordinates of the cluster maxima. Lat. = Laterality; BA = approximate Brodmann’s location; Vox. = number of significant voxels; Coordinates are in MNI space.

**Fig 4:**
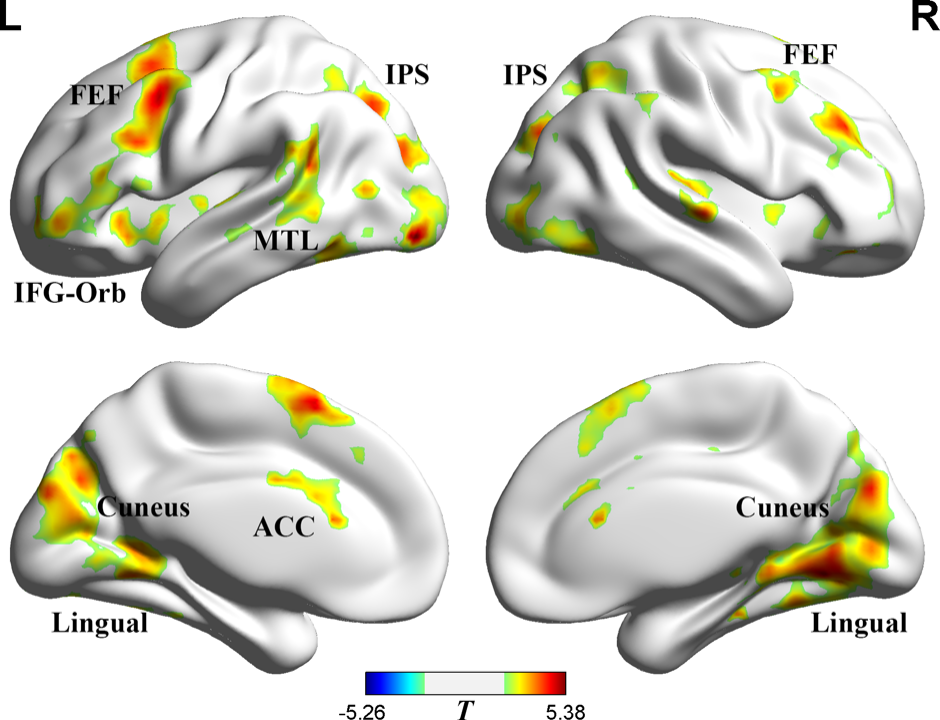
Target-switching condition > no-switching condition. Posed on medium view. Threshold: *p* = 0.05 (AlphaSim corrected). The red shows the regions that are more active in the target-switching condition.

To further identify the regions responsible for target-switching and distractor-switching, we directly compared (1) the target-switching condition vs. the no-switching condition and (2) the distractor-switching condition vs. the no-switching condition. Comparing the target-switching condition with the no-switching condition revealed significant differences in the FEF, IPS, inferior frontal gyrus (pars orbitalis) (IFG-Orb), visual cortex, middle temporal lobule (MTL), and anterior cingluate cortex (ACC), *p =* 0.05, AlphaSim corrected (Fig 4 and Table 2; target-switching condition > no-switching condition).

**Fig 5:**
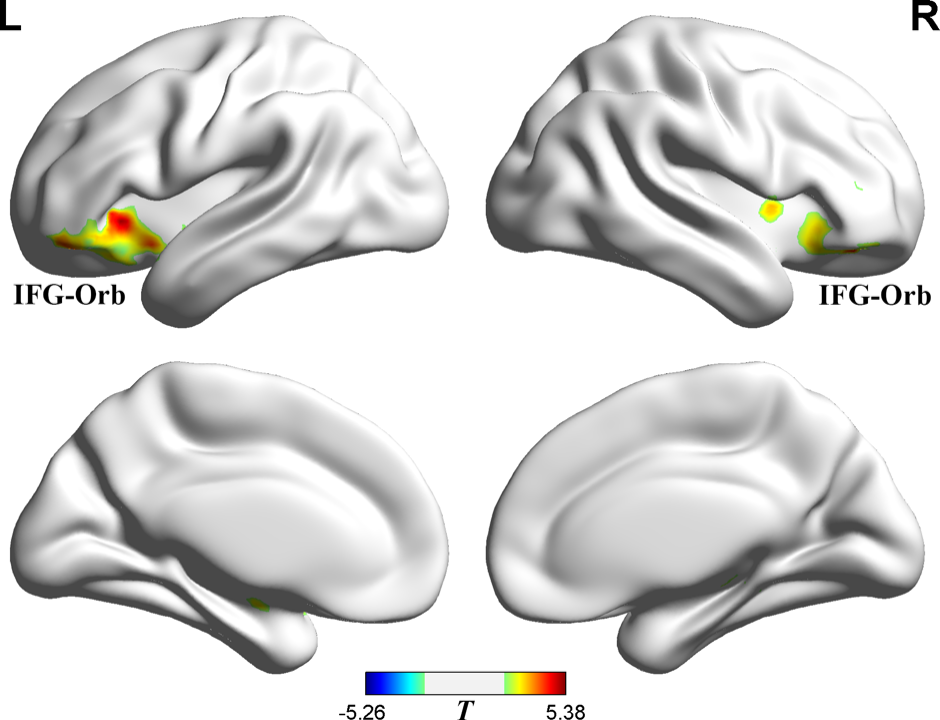
Distractor-switching condition > no-switching condition. Posed on medium view. Threshold: *p* = 0.05 (AlphaSim corrected). The red shows the regions that are more active in the distractor-switching condition.

Distractor-switching condition vs. the no-switching condition showed significant differences in the left IFG-Orb, the right IFG-Orb, *p =* 0.05, AlphaSim corrected (Fig 5 and Table 2; distractor-switching condition > no-switching condition). The IFG-Orb belongs to the ventral attention network, which has been shown to be related to bottom-up attentional control [36,37,42,46].

### ROI analysis

**Fig 6:**
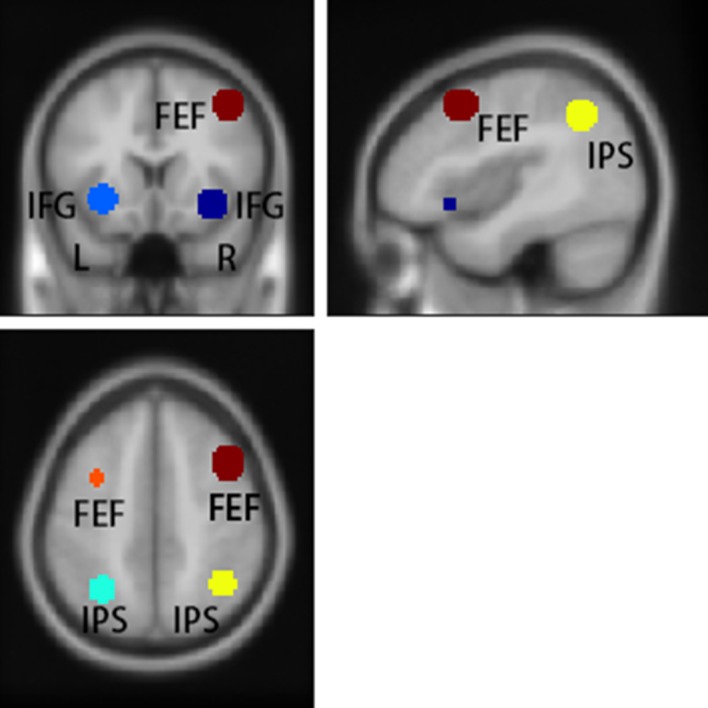
Regions of Interest (ROIs). Posed on sagittal, axial, and coronal anatomical template images.

The left FEF, right FEF, left IPS, right IPS, left IFG-Orb and right IFG-Orb were chosen from the contrast “tracking after switch” vs. “tracking before switch”, with a threshold of *p* = 0.05 (FDR corrected, two-tailed). Each ROI was defined as a sphere grown around the peak activation coordinates (See Table 3 and Fig 6), with a radius of 10 mm. The FEF and IPS regions were chosen because they are part of the dorsal attention network involved in top-down attention processes, while the IFG-Orb region was chosen because it is part of the ventral attention network, which is involved in stimulus-driven visual attention [36–41]. Meanwhile, since the identity switched in the task, we also chose the IFG-Orb region, which is part of the ventral attention network and involved in stimulus-driven visual attention [36,38,42,43].

**Table 3:** Coordinates of defined Regions of Interest (ROIs).

	Lat.	x	y	z	*T*
Inferior Frontal Gyrus (pars Orbitalis)	L	-30	21	-6	9.25
	R	33	21	-9	12.59
Intraparietal Sulcus	L	-30	-57	42	6.36
	R	39	-54	42	7.03
Frontal Eye Fields	L	-33	6	57	4.39
	R	42	15	48	4.89

Threshold: *p* = 0.05 (FDR corrected, two-tailed). x, y, and z refer to coordinates of the cluster maxima. Lat. = Laterality; Coordinates are in MNI space.

The results of contrast “tracking after switch” vs. “tracking before switch” also showed significant activation in central sulcus, supplementary motor area (SMA), ACC, middle frontal gyrus (MFG), supramarginal gyrus, Insula, Fusiform, Precuneus, *p* = 0.05, FDR corrected, two tailed (detailed results in S1 Fig and S2 Table). The activation of these regions is reported to be involved in representing visuomotor information, language perception and processing, and cognitive control [47–50].

**Fig 7:**
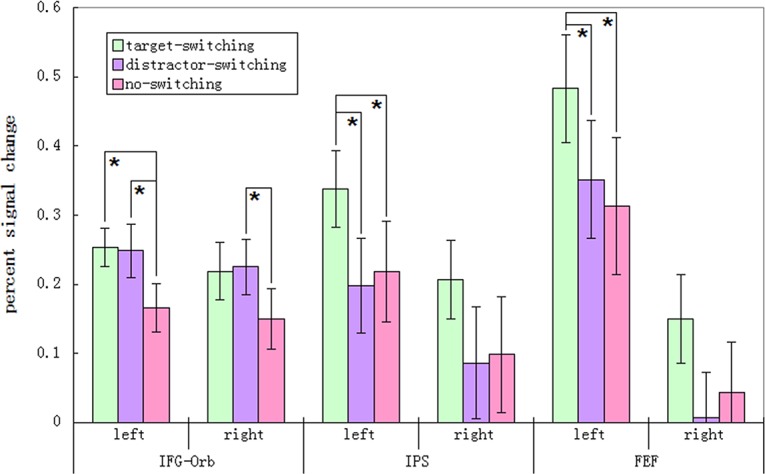
Percent Signal Change in the left IFG-Orb, right IFG-Orb, left IPS, right IPS, left FEF, and right FEF. Means ± SEM (standard errors of the mean) are shown. Asterisks represent significant differences between conditions (Bonferroni corrected).

The percent signal change data in the three experimental conditions was extracted from each ROI (S3 Table). A repeated-measures ANOVA found a significant main effect of ROIs, *F*_(5, 90)_ = 8.24, *p* < 0.001. The main effect of experimental conditions was significant, *F*_(2,36)_ = 6.68, *p* = 0.003. The interaction effect of ROIs and experimental conditions was also significant, *F*_(10, 180)_ = 3.62, *p* = 0.005. Further simple-effect analysis and pairwise comparisons were conducted to reveal the activation of the area of interest under different experimental conditions (Fig 7).

The percent signal change of left IFG-Orb was significant different between conditions *F*_(2,36)_ = 6.58, *p* = 0.004. The results showed that percent signal change in the left IFG-Orb was significantly higher in the target-switching condition than in the no-switching condition, mean difference (MD) = 0.087, *p* = 0.029. It also showed higher percent signal change in distractor-switching condition than in the no-switching condition, MD = 0.083, *p* < 0.001. But, the difference of percent signal change in the target-switching condition and in the distractor-switching condition was not significant, MD = 0.004, *p* > .99.

The main effect of the condition factor was also significant for the right IFG-Orb *F*_(2,36)_ = 4.05, *p* = 0.026. Similar to the left side, percent signal change in the right IFG was significantly higher in the distractor-switching condition than in the no-switching condition, MD = 0.076, *p* = 0.015. But the percent signal change in the target-switching condition and in the distractor-switching condition was not significantly different, MD = 0.007, *p* > .99. Also, the percent signal change difference in the target-switching condition and in the no-switching condition was not statistically significant, MD = 0.069, *p* = 0.098.

The percent signal change of the left IPS showed significant differences between conditions *F*_(2,36)_ = 7.24, *p* = 0.002. Percent signal change in the left IPS was significantly higher in the target-switching condition than in the distractor-switching condition, MD = 0.140, *p* = 0.015, and also higher than in the no-switching condition MD = 0.119, *p* = 0.044. But the percent signal change in the distractor-switching condition was not significantly different from that in the no-switching condition MD = 0.021, *p* > .99.

The percent signal change of right IPS was significantly different between conditions *F*_(2,36)_ = 4.63, *p* = 0.016. The percent signal change differences between the target-switching and distractor-switching conditions and between the target-switching and no-switching conditions showed similar trends as results in the left IPS. However, results in the right IPS were not significant. Percent signal change in right IPS showed no significant difference between pairs of experimental conditions: target-switching condition and distractor-switching condition MD = 0.121, *p* = 0.053, target-switching condition and no-switching condition MD = 0.109, *p* = 0.078, distractor-switching condition and no-switching condition MD = 0.012, *p* > .99.

The percent signal change of left FEF had significant difference between conditions *F*_(2,36)_ = 7.25, *p* = 0.002. Percent signal change of the left FEF was significantly different in the target-switching condition compared to the distractor-switching condition MD = 0.132, *p* = 0.034, and higher than in the no-switching condition MD = 0.170, *p* = 0.002. But the percent signal change in the distractor-switching condition was not significantly different from that in the no-switching condition MD = 0.039, *p* > .99.

The results also suggested that percent signal change of the right FEF was significantly different between conditions *F*_(2,36)_ = 3.83, *p* = 0.031. Although the percent signal change differences between the target-switching and distractor-switching conditions and between the target-switching and no-switching conditions showed similar trends as results in the left FEF, results in the right FEF were not significant. The percent signal change in right FEF was not significantly different from the other experimental conditions: target-switching condition and distractor-switching condition MD = 0.142, *p* = 0.070, target-switching condition and no-switching condition MD = 0.106, *p* = 0.128, distractor-switching condition and no-switching condition MD = 0.036, *p* > .99.

The ROI results are mostly consistent with the results of the whole brain analysis. The BOLD signal in the IFG-Orb changed more in the distractor-switching condition than in the no-switching condition. Meanwhile, BOLD signal in the IPS changed more in the target-switching condition than in the distractor-switching condition, and BOLD signal in the FEF changed more in the target-switching condition than in the no-switching condition.

## Discussion

This study investigated the attentional tracking of identity and locations during MIT. When targets switched identities while participants were tracking them, it required participants to rebuild the connection between targets’ identity and location. In the distractor-switching condition, participants may need to inhibit attention to the switching of the identities of the distractors. The results showed that the FEF and IPS were activated significantly more in the target-switching condition compared to the no-switching condition, and the distractor-switching condition. Finally, the IFG-Orb was significantly more active in the distractor-switching condition than in the no-switching condition.

### Attentional enhancement for target tracking

The role of the FEF is still under debate. The function of the FEF is to interact with the motor system to govern saccades. Previous studies have suggested that the FEF is responsible for eye-movement control [20,24,26]. However, in another study, researchers found evidence that suggests that the FEF is involved in attention control rather than eye-movement control [21]. Physiology studies in macaques suggest that there are two populations of cells activated in the FEF, one of which is responsible for saccades and one of which is responsible for covert shifts of attention [43,46,51]. Armstrong and colleagues [52] have suggested that the covert shifts of attention in the FEF seem to hold the location of cues during a delay interval. These findings are consistent with our finding that the FEF was significantly more active in the target-switching condition than in the no-switching condition. Muggleton and colleagues [45] delivered transcranial magnetic stimulation over the left FEF and found that the FEF modulates responses of the extrastriate cortex. Thus, we suggest the FEF plays an important role in focusing attention to targets and modulating the visual information analysis of targets.

Furthermore, researchers have suggested that the IPS helps index objects being attended to [25,26]. In addition, Howe and colleagues [26] found that the IPS responds to stationary objects. However, we found that the IPS had greater activation in the target-switching condition than in the distractor-switching condition, which indicated enhanced attention to targets after switching. Thus, we suggest that the activation of the IPS could also be responsible for attention to the targets. This interpretation fits with previous studies that found that tracking an increased number of targets increases attention load [20,21,26].

Finally, the FEF and IPS are two major parts of dorsal attention network, which is responsible for top-down control of attention in static tasks [36–41]. Therefore, in the present task, when targets switched identities during tracking, observers strengthened their attention to targets voluntarily. This voluntary attention can modulate responses of the extrastriate cortex, which could indicate updating of both identity and location in parallel [53]. Compared to previous studies, this study showed that the function of the FEF in tracking might be more related to attention control than eye-movement control and that the IPS is involved in attention tracking.

### Attentional shift and inhibition to distractors

Besides strengthening their attention to targets during tracking, it is likely that participants inhibited attention to distractors during tracking [6,7]. One study found that participants were much more likely to detect dots when the dots appeared on targets and blank areas than on distractors [7]. Other studies have shown that, the more similar distractors are to targets, the more people inhibit their attention to distractors [8]. Without requiring participants to respond to dots, Drew and colleagues [54] found that the anterior N1 component was stronger when a detection dot appeared on targets, while there was no difference when the dot was on distractors or there was no dot. In contrast, when participants were required to respond to the appearance of dots, the posterior N1 component was significantly lower than other conditions when the detection dot appeared on distractors [27].

However, the task in this study switched the identities of distractors or targets, without requiring participants to respond to the identity switching of the distractors. Thus, the significant activation in the distractor-switching condition in the IFG-Orb is related to attention control caused by the identity switch. As a part of the ventral attention network, the IFG is involved in stimulus-driven visual attention [36,38,42,43]. Fockert and Theeuwes have suggested that the IFG is involved in detecting potential distraction, but only under high load [55]. Meanwhile, other researchers have found that the IFG is activated by distractors but relatively unaffected by targets [56]. This theory is consistent with our findings.

When identities switched in the target-switching condition and the distractor-switching condition, the change is salient, and it attracts attention. The switch of the identity of the distractors seemed to attract participants’ attention in the present task, although it was not completely location-specific inhibition, which might be effective in static tasks [30]. The inhibition of distractors in the present task might occur in two stages—the first stage is captured by the change of distractors and then attention shifts to targets through task-related control.

### Comparison with previous MOT studies

In this study, participants tracked objects that switched identities in an MIT task. The results showed that the FEF and IPS (including the anterior IPS and posterior IPS) were involved in top-down control of attention to targets, while the IFG was responsible for stimulus-driven attention to changes. Previous studies have also reported that the FEF and IPS are active in these functions [20,21,26].

To study this, previous studies have manipulated the number of targets to increase attentional load in the MOT task. With this increased load, they found that the function of the MT+ (middle temporal complex) was to represent the location of moving targets [20,26]. However, the task in our study involved tracking four items. Thus, the activation of the MT+ might remain constant across the three conditions. Meanwhile, the BOLD signal in the FEF and IPS changed significantly because the attention load increased when items switched identities during tracking. Further studies should take identity-tracking load into consideration.

Previous studies have suggested that the activation of the FEF might indicate the control of eye movement [20,26] or attention tracking [21]. Furthermore, the activation of the posterior IPS might function as a spatial index or spatial tag [26] that points at the locations of attended targets, which is represented in the anterior part of the IPS [26]. However, our identity-switching task revealed that the FEF and IPS were involved in the dorsal attention network. When the identities of the targets switched, the FEF and IPS worked to strengthen goal-driven attention to targets and likely modulated the response of visual analysis in extrastriate cortex. These results may be consistent with previous studies. Since the tracking targets have no identities in MOT tasks, the activation of the FEF does not increase with the attention load of tracking items. But the activation of the IPS increases with the attention load of tracking items.

Furthermore, this study documented the neural mechanisms for attentional inhibition to distractors in the MIT task. When the identities of distractors switched during tracking, the visual change captured participants’ stimulus-driven attention. The activation of the IFG is responsible for stimulus-driven attention to the change [36,38,42,43,56]. However, results of the dot-detection task in previous studies seem to support location-specific inhibition to distractors [6–8,27,54], which might be because identity switching in this study was much more salient than the dots of previous studies, which flashed onto the screen then disappeared within 500 ms.

## Summary

This study found that paying attention to targets that switch identities increased attention load and elicited higher neural activation in the FEF and IPS. This suggests that the FEF and IPS are involved in the dorsal attention network, which helps strengthen goal-driven attention. Second, when target and distractor objects switched identities, the IFG-Orb activated when people’s attention was drawn to the change.

## Supporting Information

S1 Fig “Tracking after switch” > “tracking before switch”. Posed on medium view. Threshold: *p* = 0.05 (FDR corrected, two-tailed). The red shows the regions that are more active in the “tracking after switch”.
(TIF)

S1 Table Behavioral data. (XLS)

S2 Table Regions activated in contrast “tracking after switch” > “tracking before switch”. (DOC)

S3 Table Data of Percentage Signal Change of each ROI. (XLS)
