# Supplementary figures and images for: Brain Activation of Identity Switching in Multiple Identity Tracking Task
Source: PLoS One. 2015 Dec 23;10(12):e0145489. doi: 10.1371/journal.pone.0145489 (PMC4689547; doi:10.1371/journal.pone.0145489)

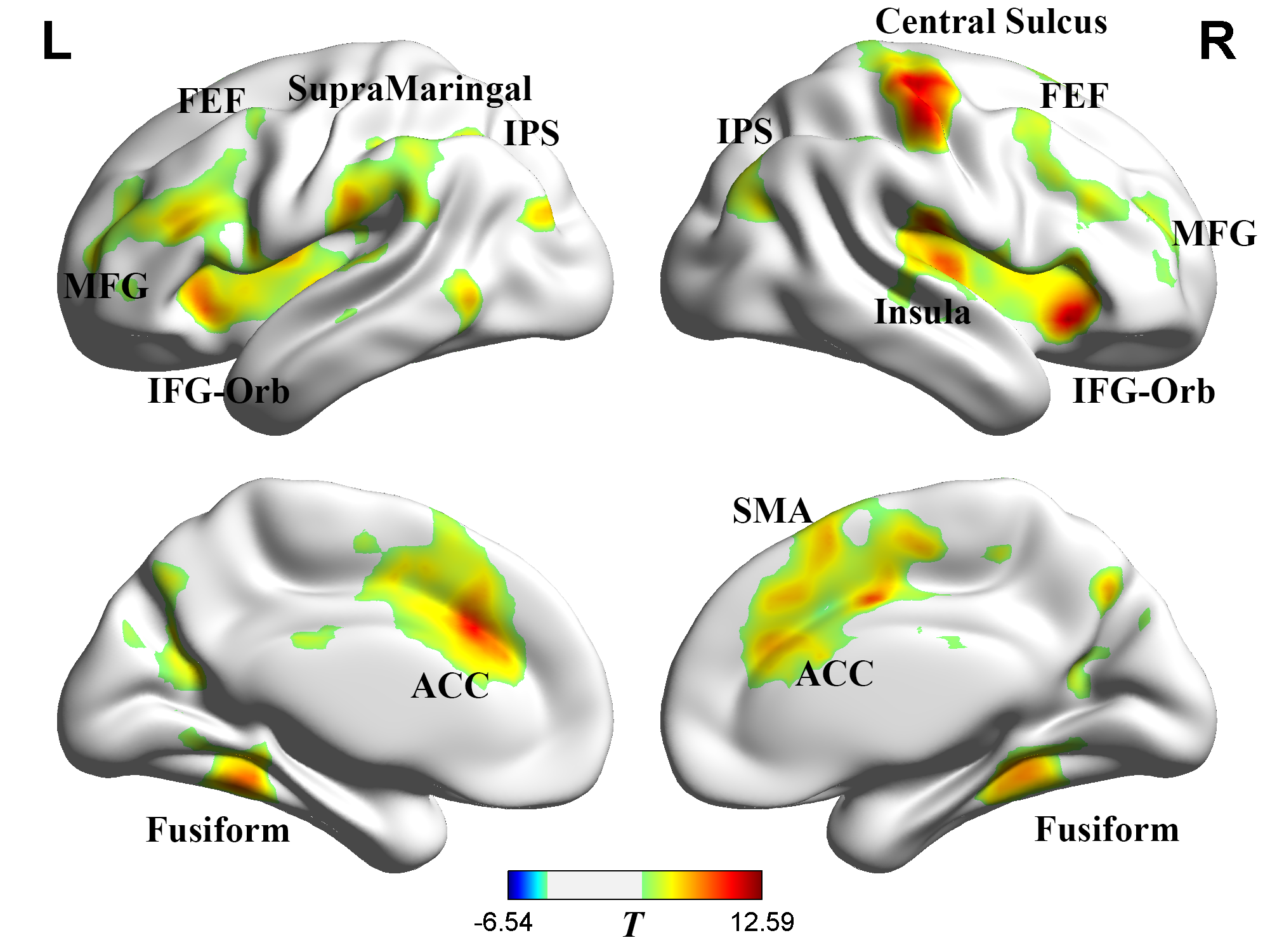

Supplement: S1 Fig — Posed on medium view. Threshold: p = 0.05 (FDR corrected, two-tailed). The red shows the regions that are more active in the “tracking after switch”. (TIF) [file pone.0145489.s001.tif]
