# Supplementary material for: Brain Activation of Identity Switching in Multiple Identity Tracking Task
Source: PLoS One. 2015 Dec 23;10(12):e0145489. doi: 10.1371/journal.pone.0145489 (PMC4689547; doi:10.1371/journal.pone.0145489)
Supplement: S2 Table — (DOC) [file pone.0145489.s003.doc]

**Table 4. Regions activated in “tracking after switch” > “tracking before switch”**

| **Region** | **Lat.** | **BA** | ***x*** | ***y*** | ***z*** | **Vox.** | ***T*** |
| --- | --- | --- | --- | --- | --- | --- | --- |
| Inferior Frontal Gyrus | L | 47,48 | -30 | 21 | -6 | 730 | 9.25 |
|  | R | 47,48 | 33 | 21 | -9 | 502 | 12.59 |
| Frontal Eye Fields | L | 6,8 | -33 | 6 | 57 | 188 | 4.39 |
|  | R | 8,9,44 | 42 | 15 | 48 | 119 | 4.89 |
| Middle Frontal Gyrus | L | 10,46 | -29 | 55 | 10 | 470 | 6.22 |
|  | R | 46,48 | 35 | 29 | 22 | 454 | 6.27 |
| Central Sulcus | R | 3,4,6 | 39 | -24 | 63 | 688 | 12.52 |
| Supplementary Motor Area | L/R | 6,24,32 | -2 | 6 | 48 | 669 | 8.26 |
| Anterior Cingulate Cortex | L/R | 24,32 | -6 | 26 | 31 | 815 | 10.72 |
| Intraparietal Sulcus | L | 7,40 | -30 | -57 | 42 | 399 | 6.36 |
|  | R | 7,40 | 39 | -54 | 42 | 181 | 7.03 |
| SupraMarginal Gyrus | L | 43,48 | -63 | -28 | 25 | 244 | 9.99 |
| Insula | L | 48 | -42 | -4 | 10 | 390 | 8.72 |
|  | R | 48 | 36 | -16 | 8 | 436 | 8.81 |
| Fusiform | L | 37 | -30 | -45 | -16 | 296 | 8.53 |
|  | R | 37 | 27 | -43 | -16 | 279 | 8.42 |
| Precuneus | L/R | 7 | 7 | -68 | 41 | 384 | 8.02 |

Threshold: *p* = 0.05 (FDR corrected, two tailed). x, y, and z refer to coordinates of the cluster maxima. Lat. = Laterality; BA = approximate Brodmann’s location; Vox. = number of significant voxels; Coordinates are in MNI space.
